# Structure Formation and Properties of Activated Supersulfate Cement

**DOI:** 10.3390/ma18091912

**Published:** 2025-04-23

**Authors:** Leonid Dvorkin, Vadim Zhitkovsky, Izabela Hager, Tomasz Tracz, Tomasz Zdeb

**Affiliations:** 1 Department of Building Elements Technology and Materials Science, National University of Water and Environmental Engineering, 33028 Rivne, Ukraine; l.i.dvorkin@nuwm.edu.ua; 2 Chair of Building Materials Engineering, Faculty of Civil Engineering, Cracow University of Technology, 31-155 Cracow, Poland; tomasz.tracz@pk.edu.pl (T.T.); tomasz.zdeb@pk.edu.pl (T.Z.)

**Keywords:** supersulfated cement, X-ray, electron microscopy, ultrasonic analysis, activation, admixture, superplasticizer, experimental–statistical model

## Abstract

The article investigates the characteristics of the phase composition and structure of supersulfated cement (SSC) during hardening using X-ray, electron microscopy, and ultrasonic analysis methods. The influence of different types of activators, hardening accelerators, and superplasticizers on the type and morphology of the newly formed phases during SSC hardening was studied. The effect of a polycarboxylate-type superplasticizer and calcium chloride on the standard consistency and setting times of SSC was experimentally determined. It was established that the introduction of the superplasticizer reduces the standard consistency by 10–16%. Experimental data showed higher effectiveness of phosphogypsum as a sulfate activator compared to gypsum stone. The strength increase of SSC at 7 days reached up to 35%, and at 28 days, up to 15%. Based on the kinetics of ultrasonic wave propagation during SSC hardening, the main stages of structure formation and the influence of cement composition on these stages were determined. The experimental results demonstrate the effect of SSC composition on its standard consistency, setting time, and mechanical properties. The impact of the type of activator and admixtures on the change in SSC strength during storage was investigated. It was found that the addition of a polycarboxylate-type superplasticizer significantly reduces the strength loss of SSC during long-term storage. Using mathematical modeling, experimentally obtained statistical models of strength were developed, which allow for the quantitative evaluation of individual and combined effects, as well as the determination of optimal SSC compositions.

## 1. Introduction

At the current stage of development in the construction industry, the use of concretes and mortars based on clinker-free or low-clinker binders is relevant for both economic and environmental reasons [1]. Such binders include supersulfated cements (SSC), which are produced from ground-granulated blast furnace slag with the addition of sulfate and alkaline activators [2,3].

The method for obtaining a binder from blast furnace slag and gypsum was first proposed in 1908 by H. Kühl [4]. According to the patent he obtained, binders can be produced based on blast furnace slag with the addition of at least 2% gypsum. The technology for such binders, which later became known as SSC, was further developed by a large group of researchers [5,6,7,8,9,10,11,12] who proposed the use of an alkaline activator–hydrated lime or Portland cement—along with gypsum, either through co-grinding the components or separate grinding followed by mixing. It was also established [12] that acidic blast furnace slags with a high content of SiO_2_ and Al_2_O_3_ and a low content of CaO are preferable for producing SSC. The compressive strength of the resulting binders at 28 days ranged from 15 to 30 MPa.

The composition and properties of modern SSC are standardized by EN 15743:2010 [13]. In accordance with this standard, the composition of SSC includes blast furnace granulated slag (≥75%), calcium sulfate (5 ≤ CS < 20%), Portland cement clinker (0 ≤ K < 5) and other additives (0 ≤ A ≤ 5%)

The mechanical and physical parameters standardized by EN 15743:2010 for SSC are presented in Table 1.

Concretes and mortars based on SSC have both advantages and disadvantages. Positive features of SSCs include their enhanced resistance in marine and sulfate environments, lactic and humic acids, and linseed oil. SSCs are characterized by relatively low heat of hydration, which makes them suitable for use in massive structures with high crack resistance. However, SSC is not recommended for use in structures exposed to alternating wetting and drying conditions, to avoid the formation of a brittle and weak surface layer, nor for concreting at low temperatures. During hardening, it is recommended that SSC-based concrete and reinforced concrete structures be kept moist for the first 2–3 weeks [14,15,16,17].

A number of studies [18,19,20,21] have focused on increasing the early strength of SSC-based concretes. The Al_2_O_3_ content in the slag has been found to be of particular importance. Slags with low alumina content require more active alkaline activation. The magnesium oxide content in the slag also plays a significant role.

The strength of SSC significantly depends on the type and amount of activating components. According to Zhou et al. [22], the amount of alkali activator generally has a more substantial influence on the hydration and strength development of SSC compared to the anhydrite content. Nguyen et al. [23] also investigated the addition of supplementary cementitious materials, such as fly ash, as an activator. The study demonstrated increased strength with fly ash content rising from 10% to 30%, which was attributed to the higher presence of alumina and the associated calcium aluminosilicate hydrates (CASH) gel formation. Moreover, when fly ash was incorporated alongside SSC replacement in the range of 15% to 50%, the initial mechanical strength of concrete was lower than that of Portland cement concrete.

Qi et al. investigated the potential enhancement of SSC by adding calcium aluminate, carbide slag, and anhydrite as activators to overcome its slow early strength development [24]. The influence of different proportions of calcium aluminate and carbide slag on SSC’s mechanical properties and hydration mechanism was examined. The results showed that using 1% calcium aluminate and 4% carbide slag as alkali activators effectively activated slag hydration in SSC, achieving a compressive strength of 9.7 MPa after 1 day.

Another study conducted by Du et al. [25] examined SSC based on solid sulfate-containing waste generated during the production of electrolytic manganese. The results indicated that, under the combined effect of cement and calcium oxide, such waste effectively activated ground-granulated blast furnace slag. The main hydration products of SSC were ettringite and calcium silicate hydrate, which contributed to the mechanical strength of composites.

Gruskovnjak et al. found that the early compressive strength of SSC using low-reactivity slag could not be improved simply by adding stoichiometric amounts of the components used to form a specific hydration product [26]. Increasing the internal dissolution rate is the best way to enhance early compressive strength.

The analysis of previous studies highlights the need for further investigation into the processes of structure formation and the properties of SSCs with different types of sulfate activators, and the additional introduction of hardening accelerators and superplasticizers. This study is aimed at exploring these issues. The purpose of the research is to identify potential reserves for improving the key properties of SSC.

## 2. Materials and Methods of Research

Granulated blast furnace slag, sulfate, and alkaline slag hardening activators, as well as additives, modifiers, hardening accelerators, and superplasticizer were used as materials for obtaining SSC. A grinding intensifier, propylene glycol, was used to increase the grinding fineness. The superplasticizer used was the additive Melflux^®^ 2651 F, a highly effective superplasticizer based on a modified polycarboxylate ester, developed by BASF (Trostberg, Germany) (hereinafter referred to as PCE).

*Granulated blast furnace slag.* The GBFS, typical in composition for Ukrainian metallurgy slag of the Kryvyi Rih Metallurgical Combine, was used in our studies. The chemical composition of the slag is presented in Table 2.

Slag basicity modulus M_b_:

(1) $$M_{b}=\frac{CaO+MgO}{SiO_{2}+Al_{2}O_{3}}=\frac{47.19+3.12}{39.51+6.47}=\frac{50}{47.6}=1.09$$

Slag activity module M_a_:

(2) $$M_{a}=\frac{Al_{2}O_{3}}{SiO_{2}}=\frac{6.47}{39.51}=0.16$$

According to the values of the activity module, slag can be attributed to the base ones with relatively low hydraulic activity [12].

The glass phase content in the slag ranged from 75% to 80%. In general, the values of M_b_, M_a_, and the glass phase content indicate the high hydraulic activity of GBFS. The alumina (Al_2_O_3_) content in the BFSC was 6.47%, significantly lower than the recommended content for SSC [27].

*Sulfate activators*. Gypsum stone and waste phosphogypsum served as sulfate activators, the chemical composition of which is given in Table 3.

For studies of sulfate activation of granulated blast furnace slags, phosphogypsum from «Rivne-Azot» (Ukraine) of long-term storage (over 10 years in dumps) was used, in which, due to natural purification processes (rain, melting snow), the content of water-soluble phosphates does not exceed 0.15%, and the content of fluorites is less than 0.4%. Such dump phosphogypsum, according to DSTU B V.2.7-2-93 (Ukrainian standard), can be attributed to conditioned PG. Before use for the manufacture of SSC, phosphogypsum was neutralized with lime milk (based on 3 wt. % CaO) with subsequent aging (storage) for 3 days and then drying at a temperature from 60 to 80 °C.

The specific surface area of phosphogypsum for different samples ranged from 260 to 330 m^2^/kg, according to Blaine’s method. The results of sedimentation analysis showed the material’s polydispersity with a predominant content of grains of the fraction 0.1–0.4 mm.

Portland cement was used as an alkaline activator. Portland cement CEM I 42.5 with the chemical and mineralogical composition of clinker, %—CaO, 66.25; SiO_2_, 22.38; Al_2_O_3_, 5.26; MgO, 0.63; Na_2_Oe, 0.21; C_3_S, 57.09; C_2_S, 21.22; C_3_A, 6.86; and C_4_AF, 12.20—was used for the research.

The main technical properties of Portland cement are as follows: specific surface area Blaine’s method, 305 m^2^/kg; standard consistency, 26.4%; setting time (hours and minutes): initial, 1–55, final, 3–25; standard strength, MPa after 28 days; bending, 6.8; compression, 51.7.

The compressive strength of mortars was determined using the standard methods according to EN 1015-11 [28]. The results obtained showed a high homogeneity of the feature analyzed. Each time, a set of three/six measurements was analyzed for homogeneity. Partial results that differed from the average by more than 10% were eliminated. An additional measurement was then taken, followed by another homogeneity verification. This process was repeated until the homogeneity of the set of results across the sample series was within ±10% of the mean value.

Experiment planning was mathematically performed using three-level plan B3 [29,30].

This method allows experiments to be conducted using an optimal design matrix and statistical processing of test results to obtain accurate experimental–statistical models in the form of polynomial dependencies, as shown in Formula (3).

(3) $$y=b_{0}+\sum_{i=1}^{k}b_{i}x_{i}+\sum_{i=1}^{k}b_{ii}x_{i}^{2}+\sum_{i,j=1}^{k}b_{ij}x_{i}x_{j},$$

where

*y* is the initial parameter;

*b*_0_, *b_i_*, *b_ii_*, and *b_ij_* are the regression coefficients;

*x_i_* and *x_ij_* are the investigated factors;

*k* is the number of factors.

The regression coefficient values provide information regarding the effect of appropriate factors on the initial parameter or property.

The algebraically calculated quantitative assessments of the coefficients of the equations were subjected to statistical analysis [30]. At the first stage of regression analysis, the standard deviation of the initial parameter and mean quadratic errors of models’ estimation coefficients are obtained. The coefficients are valuable if the design value of the Student’s t criterion is more than the given one. If a coefficient is not important, it can be omitted without re-calculating other coefficients. After the importance of the coefficients is estimated, the equation’s adequacy is checked by calculating the adequacy dispersion, the design value of Fischer’s criterion (F criterion) (F_c_), and comparing the last with a given one. The given value of the F criterion (F_t_) is obtained depending on the confidence probability (importance level) of 95% and the number of degrees of freedom. The equation is adequate for the given probability level if F_c_ > F_t_.

Based on the results obtained, regression equations and graphical relationships were constructed, both two-dimensional and three-dimensional (response surfaces).

To study the structure changes of hardened SSC stone under the influence of modifier additives, X-ray phase analysis (DRON-3 diffractometer) and electron microscopic analysis were used. Using optical microscopy in transmitted and reflected light, we also studied the features of the structure formation of binders during the hardening process.

The specific surface area of the materials was investigated using the air permeability method on the Blaine apparatus.

The ultrasonic method was used to study the kinetics of the structure formation of the SSW over time [31]. The propagation velocity of ultrasonic waves (V_us_) was determined on the UK-10P device at a frequency of 60 kHz by the following formula:

(4) $$V_{us}=lt$$

where l is the sounding base (25 mm), and t is the time of ultrasound passage through the sample.

## 3. Results and Analysis of Test Results

### 3.1. Structure Formation of the SSC

**Figure 1:**
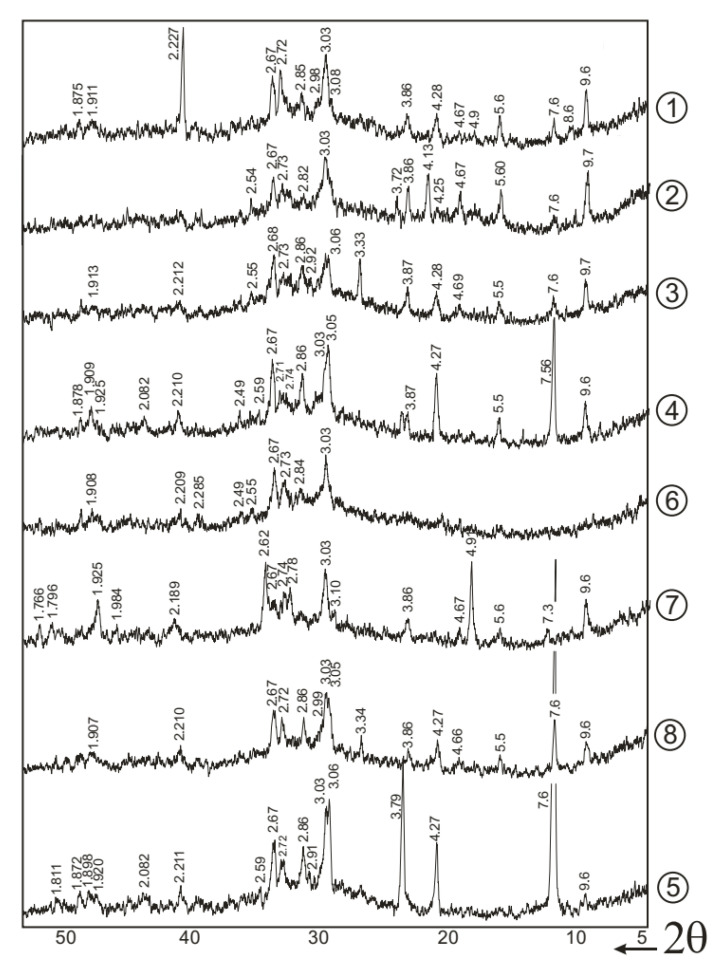
X-ray images of hardened binder: 1–8 compositions and duration of hardening of SSC according to Table 4.

To study the composition of hydrated SSC newly formed phases and the duration of hardening on them, X-ray phase studies of the hydration products of the binder were performed (Figure 1). The compositions used and the duration of storage of samples in the air are shown in Table 4.

All the above radiographs contain characteristic lines related to dihydrate gypsum CaSO_4_·2H_2_O, the low-sulfate form CASH 3CaO·Al_2_O_3_·CaSO_4_·12H_2_O and the high-sulfate form 3CaO·Al_2_O_3_·3CaSO_4_·32H_2_O (ettringite). Calcium hydrosilicates, tobermorite gel, due to X-ray amorphousness, are barely visible on radiographs.

The presence of lines of dihydrate gypsum, CaSO_4_·2H_2_O, in X-ray diffraction patterns 1 and 2 in Figure 1 indicates that even after 180 days of hardening, it was not entirely chemically bound into hydrosulfoaluminates. However, the intensity of the gypsum lines (7.56; 4.27; 3.79; 3.059 Å) significantly decreased, which indicates a decrease in its amount due to chemical binding into hydrosulfoaluminates during the hardening of SSC.

The lines characteristic of the low-sulfate form of CASH (8.92; 4.46; 3.99; 2.87; 2.45; 2.41; 1.82 Å) are more clearly distinguished in X-ray diffraction pattern No. 2, which indicates partial decomposition (recrystallization) of the high-sulfate form of CASH (9.73; 5.61; 4.69; 3.88; 2.564; 2.209; 2.154 Å) into the low-sulfate form during long-term storage of samples of hardened SSC, which, in general, coincides with the existing data [26].

Increasing the density of the hardened SSC and a certain change in the hydration conditions of the binder (compressed conditions) due to the use of the superplasticizer PCE, as well as intensification of structure formation due to the use of a hardening accelerator, CaCl_2_ (2%), led to a significant increase in the intensity of the lines of tobermorite-like calcium hydrosilicates (3.07; 2.80; 1.83; 1.67 Å) and a slight decrease in the intensity of the lines of hydrosulfoaluminates. Thus, a sharp increase in the strength of SSC when using superplasticizers is caused not only by a reduction of the porosity of the hardened stone but also by the rise in the proportion of tobermorite-like calcium hydrosilicates in the products of SSC hardening.

Increasing the content of phosphogypsum and gypsum stone from 10 to 20% led to an increase in the intensity of the lines of the content of chemically unbound dihydrate gypsum (7.56; 4.27; 3.79; 3.059 Å).

For comparison, the X-ray diffraction pattern 7 in Figure 1 presents the study results of the factory-made BFC closest in composition to SSC–BFC, which contains about 30% clinker, i.e., six times more than in SSC. A significant difference in this X-ray diffraction pattern is the presence in the binder of noticeable amounts of portlandite (4.93; 2.63; 1.93; 1.79 Å), which has not yet wholly bound into hydrosilicates after 7 days of hardening. In addition, this X-ray diffraction pattern contains lines of ettringite, hydrosilicates and dihydrate gypsum.

Electron microscopic studies were carried out using the replica method on a REMMA-101-02 electron microscope manufactured by SEMI with copper or graphite samples sputtering onto the chipped surface.

**Figure 2:**
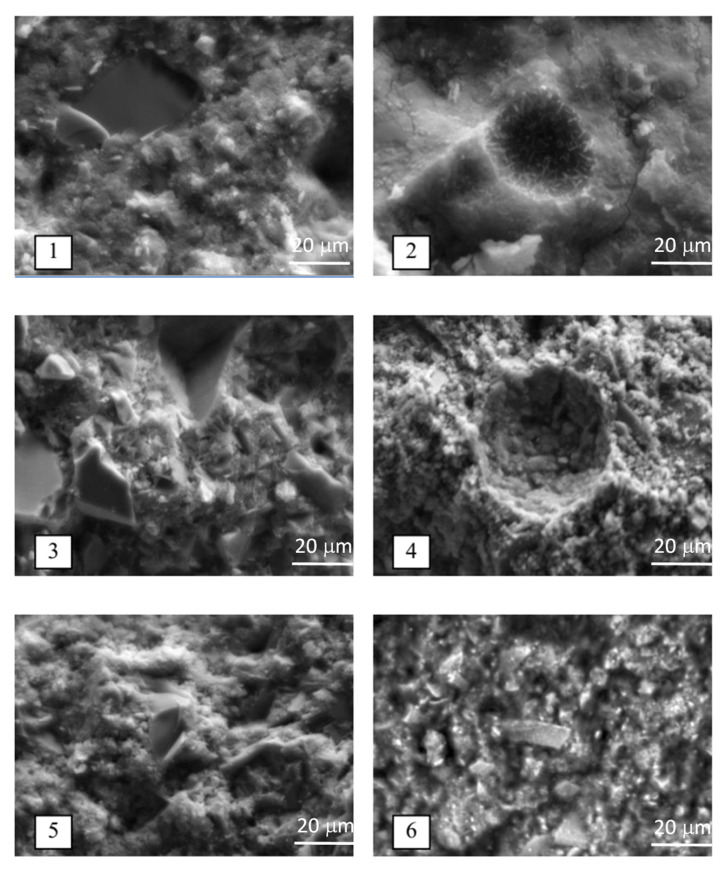
Microphotographs of different sections of hardened SSC paste (magnification ×1000): 1—BFGS (85%) + PG (10%) + PC (5%) at days; 2—GBFS (85%) + PG (10%) + PC (5%) at 180 days; 3—BFGS (82.6%) + PG (10%) + PC (5%)+ PCE (0.4%) + CaCl_2_ (2%); 4—BFGS (88%) + PG (10%) + CaO (2%); 5—BFC (100%); 6—BFGS (85%) + GS (10%) + PC (5%).

Comparison of electron microscopic images (Figure 2) of samples of hardened SSC (composition, wt.%: BFGS—85, FG—10, PC—5) after 7 and 180 days of hardening indicates an increase in the number of needle-shaped and prismatic ettringite crystals and a decrease in the content of plate-shaped crystals of dihydrate gypsum (prisms, elongated plates, intergrowths) on the chips of hardened SSC with an increase in the duration of hardening of the binder. At the same time, the disappearance of signs of the presence of portlandite is observed, for example, smooth surfaces and thin plates, which are clearly visible in samples that hardened for 7 days. Signs of the presence of monosulfoaluminate (slightly distorted hexagonal plates) can be seen in Figure 2(5). These results are in good agreement with the data of the X-ray phase analysis (Figure 1).

The use of superplasticizer PCE, as well as hardening accelerator—CaCl_2_ (2%)—(Figure 2(3)) in a certain way influenced the morphology of new formations of hardened SSC stone after 7 days. Prismatic structures are clearly visible, which can be attributed to dihydrate gypsum, as well as hydrosulfoaluminate.

Fibrous structures, which are clearly visible at 1000× magnification, probably indicate the presence of significant amounts of low-basic calcium hydrosilicates.

The increase in the content of phosphogypsum from 10 to 15% led to an increase in the signs of the content of chemically unbound dihydrate gypsum (prisms, elongated plates, intergrowths), which was noted above on the example of radiographs.

Replacing Portland cement with lime as an alkaline activator of SSC did not significantly change the morphology and composition of the bulk of the newly formed phases after 7 days.

Replacing phosphogypsum with ground gypsum stone in the same amount—10%—(Figure 2), resulted in the composition of the SSC hardening products that was practically the same as when using phosphogypsum, which indicates the similarity of the hardening processes in both cases. Increasing the duration of hardening of SSC of such a composition to 180 days had, in general, the same consequences as in the case of using phosphogypsum as a sulfate activator of increasing the number of prismatic crystals, which can be attributed to ettringite and, at the same time, the practical disappearance of signs of the presence of portlandite signs.

In the case of the composition of GBFS–water, even after long-term storage for 180 days under normal conditions (temperature 18–20 °C, humidity 90–100%), there are no noticeable signs of hydration hardening processes. Only an amorphous mass of slag glass with the inclusion of crystalline minerals, the total volume content of which does not exceed 10%, can be distinguished.

Electron microscopic studies of hardened BFC paste were also performed for comparison purposes. It can be noted that there are almost no noticeable signs of portlandite, and such crystalline newly formed phases as dihydrate gypsum and hydrosulfoaluminates are also barely noticeable (Figure 2(5)), which indicates fundamental differences in the processes of structure formation. Newly formed Portland slag cement and SSC phases are represented almost exclusively by jelly-like low-basic calcium hydrosilicates.

Using the ultrasonic pulse method, the propagation velocity of the leading edge of the ultrasonic wave (V_us_, m/s) through the hardened stone SSC was determined. To measure the propagation time of ultrasound, through sounding was used, with the sensors installed on opposite sides of the material.

**Figure 3:**
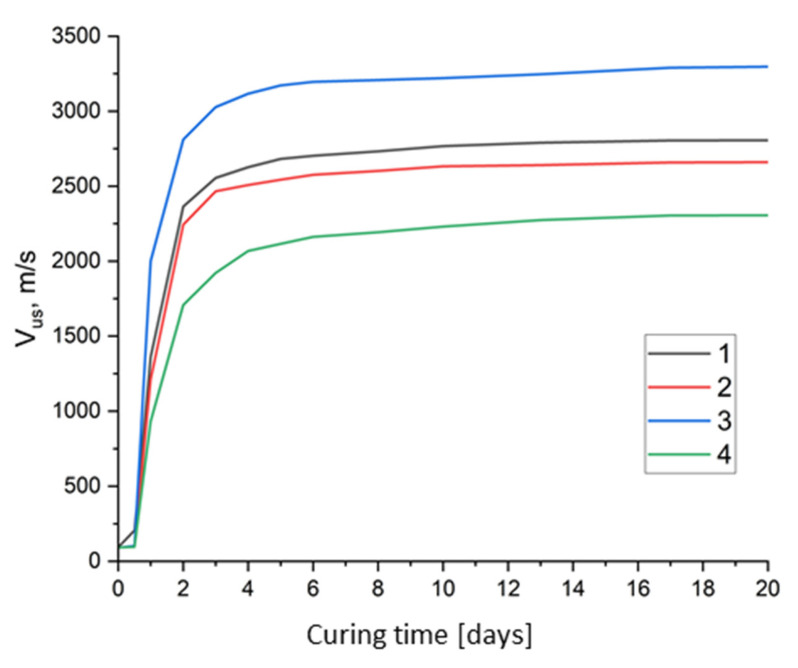
Relationship between ultrasound velocity (V_us_, m/s) in SSC paste of standard consistency and curing times: 1—W/C = 0.26 (SSC with 10% gypsum); 2—W/C = 0.28 (SSC with 15% gypsum); 3—W/C = 0.22 (SSC with 10% gypsum; 0.4% PCE + 2% CaCl_2_); 4—W/C = 0.26 (SSC with 10% gypsum stone).

The nature of the curves of the change in the speed of ultrasound transmission (Figure 3) allows us to trace all the main stages of the formation of the structure of hardened stone SSC: the initial induction period, the period of growth and fusion of gel-like calcium hydrosilicates and the crystalline newly formed phases (hydrosulfoaluminates), and the period of final strengthening of the structure with subsequent recrystallization of newly formed phases. Analyzing the obtained curves, it should be noted that the end of the induction period (the transition of the horizontal section to the vertical) is clearly visible, which practically coincides with the time of the final SSC setting by Vicat. The final setting time of the SSC is associated with the material composition and the presence of additives used during mixing. The hardening time increases significantly when using a complex modifier additive (0.4% PCE + 2% CaCl_2_). The end point of hardening is the starting point; from it there is a rapid increase in the speed of ultrasound transmission, and, accordingly, the strength to certain constant values, which depend on the type of additive and W/C.

Among the SSC compositions with sulfate activator, phosphogypsum, the slowest structure formation is observed for SSC with an excess content of phosphogypsum (15%) (Figure 2(6)), which is characterized by the highest water demand of the dough (28%). Optimization of the FG content (10%) leads to a significant intensification of hardening, and reducing the water demand of SSC through the use of the superplasticizer PCE and the hardening accelerator CaCl_2_ contributed to achieving the highest ultrasound speed, and, accordingly, the strength of SSC.

The lowest strength, and, accordingly, the speed of ultrasound transmission through the hardening binder, are characteristic of SSC using gypsum stone as a sulfate activator, which is entirely consistent with the results of studies on the strength of SSC.

The graphs in Figure 3 clearly demonstrate that, during the first three days of hardening, most SSC compositions gain more than 50% of their strength, and after seven days of hardening, more than 65%. Subsequently, the strength of the samples increases quite slowly.

### 3.2. Main Properties of Activated SSC

Properties of the SSC were studied using the following sulfate and alkaline activators and admixtures: PCE and CaCl_2_.

For SSC without superplasticizer additives, the standard consistency (SC) [32] value is about 26%, and when using the superplasticizer PCE, it is significantly reduced. The introduction of the PCE additive slightly prolongs the start of setting and, at the same time, shortens the final setting time by reducing the water demand of SSC and the porosity of the hardened binder, and this effect is enhanced with increasing admixture consumption. The CaCl_2_ accelerator admixture shortens the terms of both the initial and final setting time (Table 5).

Adding 2% of the accelerator admixture CaCl_2_ into the composition of the SSC, even at W/C = 0.40, allowed the strength to increase to more than 51 MPa at 28 days. When the CaCl_2_ consumption was increased to 3%, the strength increased to more than 61 MPa. With the joint action of the additives CaCl_2_ (2%) and PCE at W/C = 0.35, the strength increased to more than 66 MPa.

An essential indicator of SSC’s construction and technological properties is preserving standard strength during long-term storage. It is known that even under favorable storage conditions, ordinary cements are affected by CO_2_ and water vapor contained in the air [27]. At the same time, hydrated compounds and CaCO_3_ are formed on the surface of cement particles, which leads to a significant decrease in cement strength.

**Figure 4:**
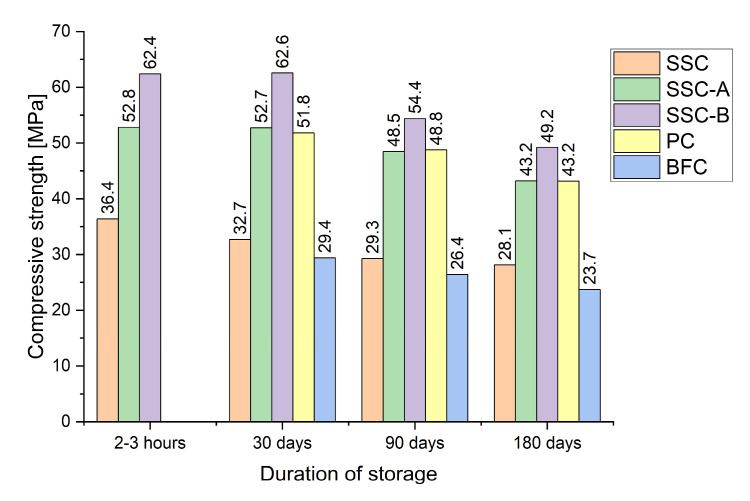
Compressive strength after 28 days with the duration of previous storage of the binder.

In our experiments to study this phenomenon on low-alumina SSC, we used SSC of optimal composition without additives of accelerators and plasticizers with a standard strength of 35 MPa (composition, %: BFGS, 85, FG, 10, PC, 5; S_p._ = 615 m^2^/kg), as well as SSC with additives PCE and CaCl_2_, and sand with M_f_ = 1.95. Samples measuring 40 × 40 × 160 mm with a binder-to-sand ratio of 1:3 were produced by molding on a laboratory vibrating platform with a vibration frequency of 3000 rpm and an amplitude of 0.35 mm for a vibration duration of 3 min. Samples were produced from the binder obtained immediately after manufacturing (grinding), after 2–3 h, and after 30, 90 and 180 days. The manufactured beam samples were kept under normal conditions (temperature 18–20°C, humidity 90–100%) before testing for compressive strength. Samples based on Portland cement CEM II 42.5 and BFC CEMIII 32.5 were also tested for comparison. The results of the studies are presented in Table 6 and Figure 4.

According to the data obtained, the rate of activity decrease during long-term storage for SSC without additives is somewhat higher (by 3–5%) than for PC and BFC. However, even when stored for 180 days, such SSC still retains a strength of about 30 MPa.

For SSC with PCE and CaCl_2_ additives, no noticeable decrease in activity was observed during the first 30 days of storage in bags, which can be explained by the temporary passivating effect of additives covering the surface of the binder particles. Subsequently, a gradual decrease in the strength of SSC with additives is observed, at approximately the same rate as for industrial cements. At the same time, the strength of SSC remains high even during long-term storage for 180 days, about 40 MPa and 50 MPa, respectively (Table 6).

The obtained results, compared with the known data on preserving the standard strength of SSC based on high-alumina GBFS [12], indicate that the SSC we proposed loses strength at approximately the same rate without additives. However, SSC with PCE and CaCl_2_ additives even have a particular advantage because these binders barely lose strength during the first 30 days of storage.

**Figure 5:**
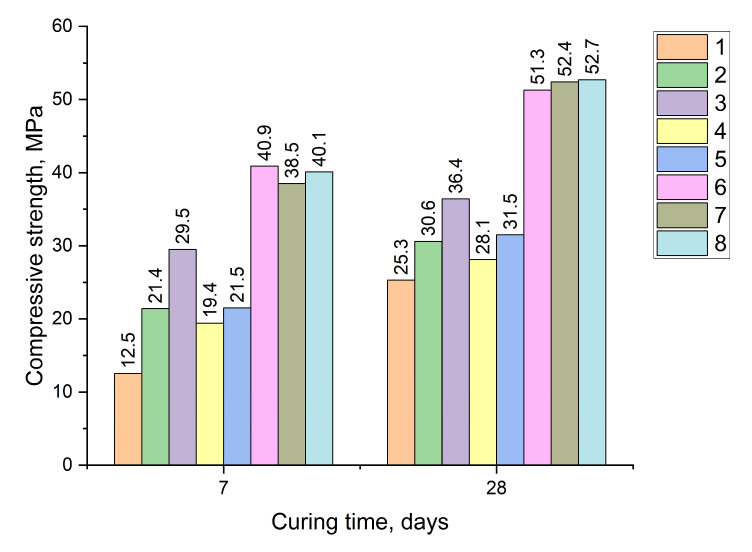
The influence of grinding fineness in combination with other factors on strength SSC (designation according to Table 7).

The strength of SCC and other factors depends on the grinding fineness, the type and content of the sulfate activator, and other modifying admixtures (Table 7 and Figure 5).

Analysis of the obtained results shows that the use of low-alumina slags, as well as phosphogypsum (PG) as a sulfate activator, combined with complex activation involving a hardening accelerator and a polycarboxylate-type superplasticizer, significantly increases early strength (up to 38–40 MPa on day 7, Figure 5). This is nearly twice the strength specified by the EN standard [13]. These values are higher than those reported by other researchers [15]. Similar results were obtained using carbide slag and sodium sulfate as activators [24].

From an economic point of view, the effectiveness of complex activation using PG, along with the accelerator and superplasticizer, is unquestionable. The choice of PG as an activator is due to its availability in waste dumps and its low utilization cost [7,12]. Calcium chloride is also one of the cheapest hardening accelerator additives; thus, the economic viability of its use is evident.

The combined effect of the addition of the hardening accelerators CaCl_2_ and CaF_2_ and PC was studied by performing algorithmized experiments in accordance with the plan B_3_ [30]. The conditions for planning experiments, the matrix, and the results of the studies on the bending (R_b_) and compression (R_c_) strength of samples at 28 days are given in Table 8 and Table 9. Based on the experimental data, regression equations for SSC’s bending and compressive strength at 28 days were obtained. The values of W/C in the experiments were determined from the condition of ensuring the standard cone flow.

**Figure 6:**
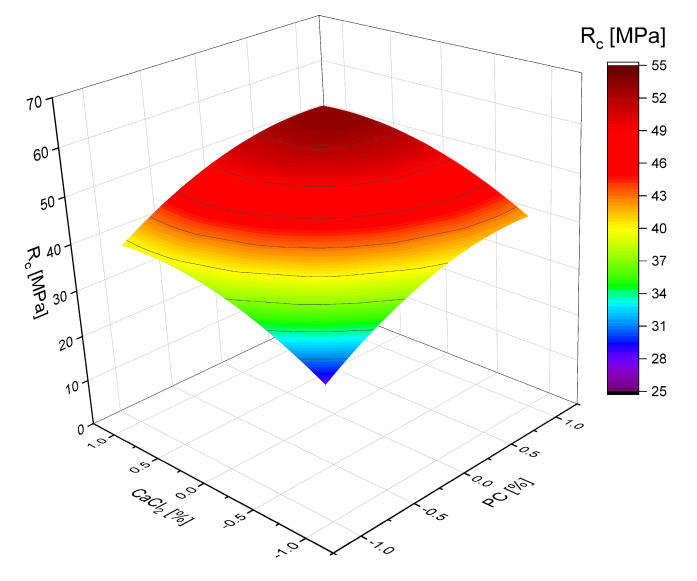
Relationship between the strength of sulfate–slag binders and the consumption of CaCl_2_ (x_2_) and PC (x_1_), the content of the additive CaF_2_ = 1% (x_3_ = 0).

**Figure 7:**
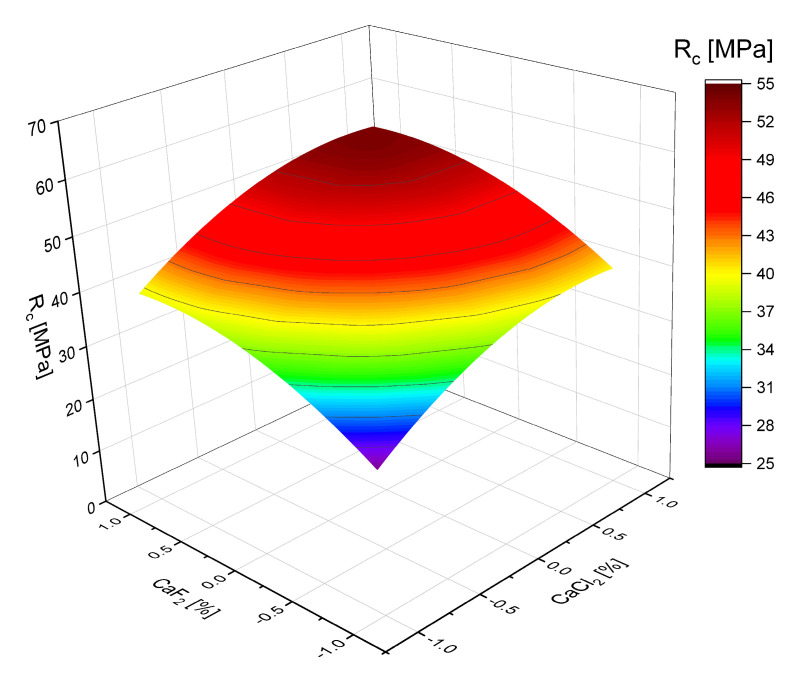
Relationship between the strength of sulfate–slag binders and the consumption of additives CaF_2_ (x_3_) and CaCl_2_ (x_2_), lime content 1% (x_3_ = 0).

Analysis of the obtained regression equations (Table 10) and graphical relationships (Figure 6 and Figure 7) indicates that all three factors affect the strength of SSC positively, that is, as their values increase, their strength increases, and by their influence on strength they can be arranged in the following series: X_3_ > X_2_ > X_1_.

The influence of the studied factors on strength is more pronounced in the case of compressive strength, while the bending strength changes relatively little. The effect of slag activation with fluorite, which consists of activating the surface of its particles and, thus, increasing their reactivity, gives a more pronounced effect than the effect of increasing the concentration of Ca^2+^ ions when using calcium chloride.

## 4. Conclusions

1. The hardening process of SSCs based on low-alumina slag involves the formation of both low- and high-sulfate calcium aluminosilicate hydrates (CASH) and calcium hydrosilicates. CASH phases predominantly develop during the first 3–7 days, while calcium hydrosilicates form in the later stages of hydration.
2. The use of a hardening accelerator in combination with a polycarboxylate-based superplasticizer significantly enhances the hydration kinetics of SSCs, resulting in notable increases in both early-age and 28-day compressive strength.
3. The type of activator used in SSCs strongly influences the crystallinity and morphology of the hydration products. Over time, the quantity of needle-like and prismatic ettringite crystals increases, while the content of plate-like calcium sulfate dihydrate diminishes. The incorporation of superplasticizer and accelerator promotes the development of a fibrous microstructure dominated by low-basicity calcium hydrosilicates. However, increasing the phosphogypsum content beyond 10% leads to a greater amount of unbound calcium sulfate dihydrate.
4. Analysis of ultrasonic pulse velocity profiles reveals the key structural development stages of SSCs: an initial induction period, a growth phase of gel-like crystalline formations, and a final stage involving solid structure formation and recrystallization.
5. Experimental data show that the rate of strength loss in SSCs during storage is composition-dependent. SSCs without accelerators or superplasticizers tend to lose strength more rapidly than Portland or slag-Portland cement. In contrast, SSCs modified with calcium chloride and polycarboxylate ether (PCE) retain their standard strength during the first 30 days of storage.
6. Polynomial regression models developed for SSC compressive strength demonstrate the individual and synergistic effects of calcium chloride, PCE, and Portland cement content. These models confirm the positive contribution of these components to both compressive and flexural strength.

## Figures and Tables

**Table 1:** Mechanical and physical requirements given as characteristic values (EN 15743:2010 [13]).

Strength Class	Compressive Strength, MPa				Initial Setting Time, min
	Early Strength		Standard Strength		
	**2 Days**	7 Days	28 Days		
32.5 L	-	≥12.0	≥32.5	≤52.5	≥75
32.5 N	-	≥16.0			
42.5 L	-	≥16.0	≥42.5	≤62.5	≥60
42.5 N	≥10.0	-			
52.5 L	≥10.0	-	≥52.5	-	≥45
52.5 N	≥20.0	-			

**Table 2:** Chemical composition of granulated blast furnace slag.

Oxide Content in Slag, wt. %								
SiO_2_	Al_2_O_3_	Fe_2_O_3_	CaO	MgO	SO_3_	MnO	LOI	Σ, %
39.51	6.47	0.14	47.19	3.12	1.76	1.14	0.59	99.92

**Table 3:** XRF chemical composition of gypsum and phosphogypsum (% wt.).

Gypsum (Stone) (GS)								
LOI	SiO_2_	Al_2_O_3_	Fe_2_O_3_	CaO	MgO	SO_3_	CaSO_4_·2H_2_O	
17.87	8.54	0.70	0.4	29.88	0.41	41.85	89.97	
Phosphogypsum (PG)								
CaO	SO_3_	P_2_O_5_ total	P_2_O_5_ water-soluble.	Fe_2_O_3_	Al_2_O_3_	F	MgO	Cl
38.3	59.1	0.69	0.04	0.16	0.34	0.14	0.004	0.01

**Table 4:** SSC compositions and curing times.

No.	SSC Composition,%				Hardening Duration, Days
	BFGS	Sulfate Component	Portland Cement	Additive	
1	90	PG-10	PC-5	-	7
2	90	PG-10	PC-5	-	180
3	90	PG-10	PC-5	PCE—0.4%, CaCl_2_–2%	7
4	85	PG-15	PC-5	-	7
5	80	PG-20	PC-5	-	7
6	90	GS-10	PC-5	-	7
7	BFC				7
8	80	GS-20	PC-5	-	180

Note: PG—lime-neutralized phosphogypsum, GS—gypsum stone, PC—Portland cement, BFGS—blast furnace granulated slag, BFC—Blast furnace cement.

**Table 5:** Standard consistency and setting times of SSC.

SSC Composition,%			Admixture, %	Standard Consistence, %	Setting Time, h-min	
BFGS	Sulfate Component	Alkaline Component				
85	PG-10	PC-5	-	26	3–10	7–40
85	GS-10	PC-5	-	26	3–30	7–50
88	PG-10	Lime-2	-	26	3–50	8–10
88	GS-10	Lime-2	-	26	3–50	8–20
85	PG-10	PC-5	PCE—0.2%	24	4–20	7–40
85	PG-10	PC-5	PCE—0.4%	22	4–40	7–10
85	FG-10	PC-5	PCE—0.4% + CaCl_2_–2%	22	3–20	6–50
85	GS-10	PC-5	PCE—0.4%	22	4–50	8–20

Note: PG—lime-neutralized phosphogypsum, GS—gypsum stone, PC—Portland cement.

**Table 6:** Decrease in the 28-day compressive strength of SSC and similar industrial cements, depending on the duration of storage.

Type of Binder	Additive	Compressive Strength After 28 Days with the Duration of Previous Storage of the Binder							
		2–3 h		30 Days		90 Days		180 Days	
		Mean	Standard Deviation (SD)	Mean	SD	Mean	SD	Mean	SD
SSC	-	36.4	1.6	32.7	1.5	29.3	1.4	28.1	1
SSC-A	PCE-0.4%	52.8	1.4	52.7	1.8	48.5	2.0	43. 2	2.1
SSC-B	PCE-0.6% CaCl_2_-2%	62.4	1.4	62.6	1.1	54.4	1.6	49.2	1.5
PC	-	-	-	51.8	1.8	48.8	1.6	43.2	2.0
BFC	-	-	-	29.4	1.1	26.4	1.2	23.7	1.6

Note: PC—Portland cement, BFC—Blast furnace cement, SSC—supersulfated cement, SSC-A and SSC-B—supersulfated cement with admixtures.

**Table 7:** The influence of grinding fineness in combination with other factors on strength SSC.

#	SCC Composition, %			Specific Surface Area, m^2^/kg	Type/Content (%) of Modifying Admixtures	Compressive Strength, MPa			
	BFGS	Sulfate Activator	PC			7 Days		28 Days	
						Mean	SD	Mean	SD
1	80	PG/15	5	390	-	12.5	1.1	25.3	1.8
2	80	PG/15	5	615	-	21.4	2.3	30.6	1.8
3	85	PG/10	5	615	-	29.5	1.6	36.4	1.4
4	90	PG/5	5	610	-	19.4	1.4	28.1	2.2
5	90	GC/10	5	620		21.5	1.4	31.5	1.3
6	90	PG/10	5	610	CaCl_2_/2	40.9	1.8	51.3	1.3
7	90	PG/10	5	615	CaCl_2_/2	38.5	2.0	52.4	1.4
8	90	PG/10	5	610	PCE/0.4	40.1	1.7	52.7	1.7

**Table 8:** Conditions for planning an experiment.

Technological Factors		Levels of Variation			Variation Interval
Natural View	Coded View	−1	0	+1	
PC content, %	*x*_1_	1.0	2.0	3.0	1.0
Content of CaCl_2_, %	*x*_2_	0	1.0	2.0	1.0
Content of CaF_2_, %	*x*_3_	0	1.0	2.0	1.0

**Table 9:** Planning matrix and research results.

Coded Factor Values			Strength, MPa			
*x*_1_	*x*_2_	*x*_3_	Bending (R_b_)		Compressive (Rc)	
			Mean	SD	Mean	SD
+1	+1	+1	11.52	0.21	59.44	0.99
+1	+1	−1	8.51	0.11	39.58	1.92
+1	−1	+1	8.93	0.38	40.62	1.53
+1	−1	−1	7.50	0.95	26.39	2.03
−1	+1	+1	9.57	0.46	43.27	1.64
−1	+1	−1	7.73	0.85	28.58	1.11
−1	−1	+1	7.76	0.41	29.24	1.86
−1	−1	−1	7.50	1.01	20.20	1.51
+1	0	0	9.97	0.66	50.23	1.55
−1	0	0	8.91	0.18	39.01	1.81
0	+1	0	10.04	0.51	51.09	1.8
0	−1	0	8.61	0.22	37.58	1.83
0	0	+1	10.12	0.32	51.00	1.62
0	0	−1	8.51	0.81	36.57	1.98
0	0	0	9.70	0.33	48.43	2.05
0	0	0	9.66	0.48	48.37	1.56
0	0	0	9.74	0.71	48.41	1.77

**Table 10:** Regression equation for the strength of SSC with additives of hardening accelerator CaCl_2_ and activator CaF_2_.

Output Parameters	Regression Equation (Confidence Probability (Importance Level) of 95%)
Bending strength SSC at 28 days, MPa	$R_{b}=9.7+0.5x_{1}+0.7x_{2}+0.8x_{3}-0.3x_{1}^{2}-0.4x_{2}^{2}-0.4x_{3}^{2}$ (5)
	Standard deviation: 0.724 Mean quadratic errors: 0.256 Criterion of Fisher (calculated): 2.83
Compressive strength of SSC at 28 days, MPa	$R_{c}=48.4+5.6x_{1}+6.8x_{2}+7.2x_{3}-3.8x_{1}^{2}-4.1x_{2}^{2}-4.6x_{3}^{2}$ (6)
	Standard deviation: 1.313 Mean quadratic errors: 0.464 Criterion of Fisher (calculated): 3.81

## Data Availability

Raw data were generated at the National University of Water and Environmental Engineering in Ukraine and Cracow University of Technology. Derived data supporting the findings of this study are available from the authors on request.
